# Differential effects of theta/beta and SMR neurofeedback in ADHD on sleep onset latency

**DOI:** 10.3389/fnhum.2014.01019

**Published:** 2014-12-23

**Authors:** Martijn Arns, Ilse Feddema, J. Leon Kenemans

**Affiliations:** ^1^Department of Experimental Psychology, Utrecht UniversityUtrecht, Netherlands; ^2^Research Institute BrainclinicsNijmegen, Netherlands

**Keywords:** ADHD, neurofeedback, theta, SMR, theta/beta, sleep, sleep onset insomnia, EEG

## Abstract

Recent studies suggest a role for sleep and sleep problems in the etiology of attention deficit hyperactivity disorder (ADHD) and a recent model about the working mechanism of sensori-motor rhythm (SMR) neurofeedback, proposed that this intervention normalizes sleep and thus improves ADHD symptoms such as inattention and hyperactivity/impulsivity. In this study we compared adult ADHD patients (*N* = 19) to a control group (*N* = 28) and investigated if differences existed in sleep parameters such as Sleep Onset Latency (SOL), Sleep Duration (DUR) and overall reported sleep problems (PSQI) and if there is an association between sleep-parameters and ADHD symptoms. Secondly, in 37 ADHD patients we investigated the effects of SMR and Theta/Beta (TBR) neurofeedback on ADHD symptoms and sleep parameters and if these sleep parameters may mediate treatment outcome to SMR and TBR neurofeedback. In this study we found a clear continuous relationship between self-reported sleep problems (PSQI) and inattention in adults with- and without-ADHD. TBR neurofeedback resulted in a small reduction of SOL, this change in SOL did not correlate with the change in ADHD symptoms and the reduction in SOL only happened in the last half of treatment, suggesting this is an effect of symptom improvement not specifically related to TBR neurofeedback. SMR neurofeedback specifically reduced the SOL and PSQI score, and the change in SOL and change in PSQI correlated strongly with the change in inattention, and the reduction in SOL was achieved in the first half of treatment, suggesting the reduction in SOL mediated treatment response to SMR neurofeedback. Clinically, TBR and SMR neurofeedback had similar effects on symptom reduction in ADHD (inattention and hyperactivity/impulsivity). These results suggest differential effects and different working mechanisms for TBR and SMR neurofeedback in the treatment of ADHD.

## Introduction

Humans spend about one third of their lives in a sleeping state, yet the function and implications of this “inactive state” are to date not fully understood, especially in relation to psychiatric problems such as depression and attention deficit hyperactivity disorder (ADHD). A well known, validated and accepted model in sleep medicine is the two-process model by Borbély (1982). This model postulates a sleep-wake dependent Process-S and the circadian Process-C. Process-S can be quantified by the build-up of Electroencephalogram (EEG) slow activity (delta and theta) during the day, often referred to as sleep homeostatic drive, and is thus a function of duration of prior waking (Achermann et al., 1993). Also, this slow EEG activity is considered the hallmark of drowsiness (Arns et al., 2010), and shows a gradual decline with subsequent sleep stages. Interestingly, this type of EEG pattern is also seen in a subgroup of ADHD patients (excess theta, or greater theta/beta ratio (Arns et al., 2013a)). Process-C can be quantified by assessing the different circadian measures such as melatonin (using the Dim Light Melatonin Onset (DLMO: Van der Heijden et al., 2005) or core-body temperature. Both Process-S and Process-C, and especially their interaction, play a crucial role in sleep-wake regulation and optimal vigilance regulation. This model also helps explain many sleep related problems, such as jetlag (by a misalignment of Process-C with Process-S) and the effects of sleep deprivation or sleep restriction (Increased sleep pressure or Process-S). Often sleep problems are regarded as a comorbidity in psychiatric disorders. However, recent studies challenge this notion and implicate a causative role in the etiology of circadian and sleep problems in for example Depression (McClung, 2013) and ADHD (Arns and Kenemans, 2014). In the following, we will focus mainly on the role of sleep in ADHD (subgroups).

### Sleep and cognition in children

In a recent large meta-analysis in 35.936 healthy children, Astill et al. (2012) demonstrated clear associations between sleep duration and executive function and school performance (positive), and between sleep duration and inernalizing and externalizing behavior (negative). In addition, a meta-analysis in 690.747 children recently confirmed that, today, children sleep 1 h and 15 min less than a 100 years ago (Matricciani et al., 2012). Interestingly, several recent studies demonstrated that when morning school-time was delayed by 25–30 min, a 29–45 min *increase* in sleep duration occurred, with subsequent reductions in daytime sleepiness, depressed mood and caffeine use (Owens et al., 2010; Boergers et al., 2014). In a recent multicenter study among 9.000 students, it was even shown that when school start times were shifted from 7.35 AM to 8.55 AM, the number of car crashes among teen drivers was reduced by 70% (Wahlstrom et al., 2014). These studies further support the above trend that children and adolescents today have a too short sleep duration, further supported by a trend for increased signs of drowsiness in healthy children across the last 10 years, as measured with the more objective Electroencephalogram (EEG) Theta/Beta ratio (Arns et al., 2013a), which can be regarded as a measure of drowsiness (as per above, reflective of Process S, or increased homeostatic sleep drive). The question arises if this trend of reduced sleep duration for children has any repercussions in daily life, and/or could possibly be associated with complaints often reported in the ADHD spectrum, given the reported relation between reduced sleep duration and impaired executive functioning and higher levels of internalizing/externalizing behavior (Astill et al., 2012), as well as attentional (Belenky et al., 2003; Van Dongen et al., 2003; Axelsson et al., 2008) and mood problems (Owens et al., 2010; Boergers et al., 2014).

### Sleep, sleep restriction and ADHD

Sleep deprivation is known to have detrimental effects on cognitive functioning. However, as was demonstrated by Van Dongen et al. (2003), a sleep *restriction* to six hours for 14 days had comparable effects on cognitive functioning (sustained attention and working memory) as two nights of full sleep deprivation, in line with predictions the authors made from the above 2-process model of sleep. Moreover, people submitted to this regimen of sleep restriction were unaware of their cognitive deficits. Similar findings have been reported after 5–7 days of sleep restriction (Belenky et al., 2003; Axelsson et al., 2008). Interestingly, these studies also showed that these cognitive impairments, most specifically inattention, took more days of normal sleep to recover than the initial sleep restriction (Belenky et al., 2003; Axelsson et al., 2008). Sleep restriction studies have also been conducted in children, albeit not as extensively as in adults. In general, sleep restriction studies in healthy children have demonstrated impairments of attention (Fallone et al., 2001, 2005; Sadeh et al., 2003; Beebe et al., 2008) and increased externalizing behavior (impaired behavioral regulation) after one week of sleep restriction (Belenky et al., 2003). Thus, core symptoms of ADHD such as inattention and externalizing behavior can be induced in healthy children through sleep restriction (Fallone et al., 2001; Golan et al., 2004), suggesting a role for sleep in the etiology of ADHD.

Several sleep disorders, such as sleep apnea and restless legs syndrome, are more prevalent in ADHD. Substantial improvements in ADHD complaints have been reported, when such specific sleep disorders were treated (for review also see (Arns and Kenemans, 2014; Cortese et al., 2013)). These sleep disorders most likely impact on Process-S, resulting in an impaired sleep homeostasis and thus sustained sleep restriction, expressed in more signs of drowsiness EEG or theta.

Other studies have investigated the occurrence of idiopathic “sleep-onset insomnia” (SOI), also called “delayed sleep phase syndrome”, in ADHD (Van der Heijden et al., 2005). The main symptom in SOI is a difficulty falling asleep at a desired bedtime and/or a sleep onset latency (SOL) of more than 30 min (Smits et al., 2001; Van Veen et al., 2010). SOI is present in 72–78% of unmedicated children and adults with ADHD and in this subgroup of patients with SOI, a delayed DLMO has been found (delayed melatonin onset), suggestive of a circadian phase delay (Van der Heijden et al., 2005; Van Veen et al., 2010). In further agreement with these findings, Rybak et al. (2007) reported that adult ADHD is characterized by a higher prevalence of “evening types”, characterized by a delayed circadian phase. Also consistent with this, Arns et al. demonstrated an association between high sunlight intensity and low ADHD prevalence, which could indicate an involvement of circadian clock disturbances Arns et al. (2013c) in ADHD etiology.

In this subgroup, a delayed Process-C causes Process-S and Process-C to intersect at a later time, thus explaining an inability to fall asleep at an age appropriate bedtime. The cause of this delayed circadian phase in ADHD has been attributed to a combination of genetic factors and environmental factors, especially evening exposure to blue-light sources such as LED lights and tablets (Baird et al., 2011; Bijlenga et al., 2011; Chaste et al., 2011; Arns et al., 2013d). Since children all have to go to school at the same time, a delayed sleep onset can cause a reduced sleep-duration and hence result in sleep restriction and associated complaints, such as inattention and/or externalizing behavior. Conversely, as noted above, when morning school times are delayed, overall improvements are seen on mood, alertness and a lower incidence of car crashes (possibly reflective of reduced inattention) (Owens et al., 2010; Boergers et al., 2014; Wahlstrom et al., 2014).

### Neurofeedback and sleep

Several studies have demonstrated that Sensori-Motor Rhythm neurofeedback (SMR) results in increased sleep spindle density during sleep (Sterman et al., 1970; Hoedlmoser et al., 2008), decreased sleep latency (Hoedlmoser et al., 2008) and increased total sleep time (Hoedlmoser et al., 2008; Cortoos et al., 2010). Research has also demonstrated that melatonin results in an increased sleep spindle density (Dijk et al., 1995) and decreased sleep latency (Van der Heijden et al., 2007), suggesting overlap in the working mechanisms of SMR neurofeedback and melatonin.

**Figure 1:**
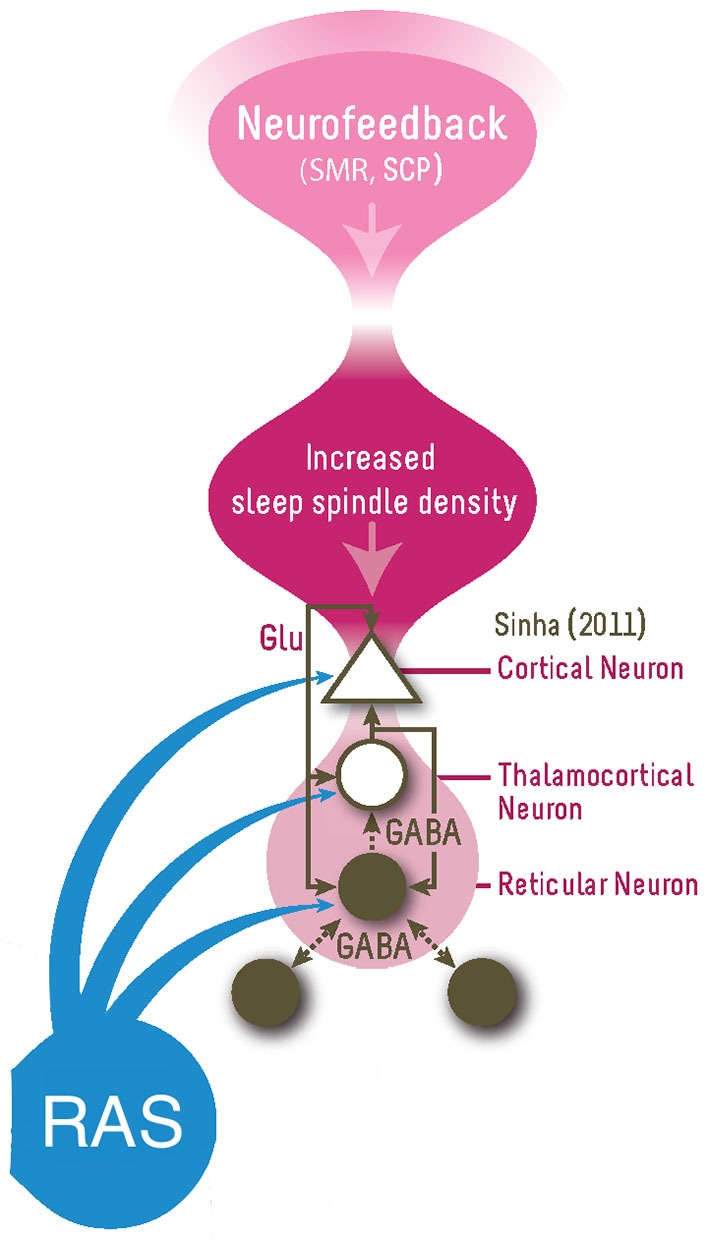
**This figure summarizes the proposed model for how neurofeedback (right top) impact on the vigilance system (responsible for sleep-wake regulation)**. SMR Neurofeedback is thought to train the Reticular-Thalamocortical-Cortical network by increasing the synaptic strengths within this network via the three-way glutamatergic (Glu) connections, resulting in long-term potentiation (LTP) which increases synaptic sensitivity and the probability of future activation in this network, namely by increased sleep spindle density during sleep (Sterman and Egner, 2006). This increased sleep spindle density results in decreased sleep latency and increased total sleep time, resulting in vigilance stabilization (or improved sleep homeostatis). The RAS (Reticular Activating System) also has an influence on this circuitry (Figure adapted from Sinha (2011)).

Sleep spindles are generated by the GABA-ergic thalamic reticular neurons and are synchronized through glutamatergic cortico-thalamic projections (De Gennaro and Ferrara, 2003). The spindle oscillation generated in the reticular neurons is transferred to thalamocortical relay cells in the dorsal thalamic nuclei through GABAergic synapses, producing inhibitory postsynaptic potentials (IPSPs) and these IPSPs travel through glutamatergic thalamocortical axons to generate rhythmic excitatory postsynaptic potentials (EPSPs) in the cortex (Sinha, 2011), also see Figure 1 for a summary. Therefore, SMR neurofeedback is hypothesized to directly train the sleep spindle circuit given the overlap in frequency and location and as evidenced by studies demonstrating an increase in sleep spindle density after SMR neurofeedback (Sterman et al., 1970; Hoedlmoser et al., 2008). It was proposed that training this network function using neurofeedback results in long-term potentiation (LTP) which increases the synaptic strengths within this network and increase the likelihood of future activation of this network (Sterman and Egner, 2006; Arns and Kenemans, 2014), which was seen as increased sleep spindle density during sleep (Sterman et al., 1970; Hoedlmoser et al., 2008).

The influence of SMR neurofeedback on sleep spindles (Sterman et al., 1970; Hoedlmoser et al., 2008), and effects of SMR neurofeedback on SOL and sleep duration have been demonstrated (Hoedlmoser et al., 2008; Cortoos et al., 2010), however this has not been reported yet in ADHD. Another well-investigated neurofeedback protocol for ADHD is Theta/Beta ratio (TBR) neurofeedback (Arns et al., 2013b), and in earlier work we had observed that patients treated with both SMR and TBR neurofeedback improved on sleep (Arns, 2011; Arns et al., 2012), however no further studies have specifically looked at the effects of TBR neurofeedback on sleep. Furthermore, the TBR neurofeedback we apply aims at training beta frequencies above the SMR band (e.g., 15–20 Hz), so a further reason for including this protocol is to investigate the specificity of training a lower beta band or SMR (12–15 Hz) vs. a higher beta band (e.g., 15–20 Hz) in relation to sleep. Alternatively, Gevensleben et al. (2012), have hypothesized that the effects of TBR neurofeedback are mainly explained by learned self-regulation over brain activity associated with attention, which suggests another working mechanism for the efficacy of neurofeedback in ADHD. Therefore, in this study we employed an open-label design based on data from our clinic where sleep parameters as well as ADHD rating scale (RS) data were collected at different time points through neurofeedback treatment (using either SMR or TBR protocols) as well as data collected in healthy controls.

The primary aims of this study thus were to (1) compare our ADHD patients to a control group in order to substantiate differences on sleep parameters such as SOL, Sleep Duration (DUR) and overall reported sleep problems (PSQI) as well as establish a correlation between sleep-parameters and ADHD symptoms; and (2) investigate the effects of SMR and Theta/Beta (TBR) neurofeedback on ADHD symptoms, sleep parameters such as SOL, DUR and PSQI score and investigate if these sleep parameters mediate treatment outcome. We hypothesize that both SMR and TBR will demonstrate similar improvements on sleep parameters (SOL and DUR) and that these improvements mediate clinical improvement on inattention and hyperactivity/impulsivity. In addition in our analysis we will test for differential effects of protocol.

## Methods

### Participants

This study is an open-label pilot study. Twenty-eight healthy controls (age: 21–64 yrs.; 13 male) and 51 patients with ADHD (age: 6–53 yrs; 35 male; 32 children) were included in this study. All files from patients seen in our clinic (Psychology Practice Brainclinics, Nijmegen, The Netherlands) between August 12th 2008 and December 4th 2013 were screened (The patients reported here overlap with the patients reported earlier by Arns et al. (2012)). Patients were screened for ADHD or ADD by a clinical psychologist using a structured interview (MINI Plus Dutch version 5.0.0, for adults or MINI KID for children) during intake. For inclusion in this study all data were screened and inclusion was based on DSM 5 criteria (American Psychiatric Association, 2013). During intake, every 10th session and outtake a self-report scale for ADHD symptoms (Kooij et al., 2005) was assessed (with a maximum score of 9 per sub-scale), as well as a self-report scale for quality of sleep (Pittsburgh Sleep Quality Index (PSQI); (Buysse et al., 1989) that also included questions about SOL and sleep duration (DUR). Only subjects with a primary diagnosis of ADHD/ADD were included in the study. All patients signed an informed consent form before treatment was initiated.

### Controls

Twenty-eight healthy adult controls were included between August 31st 2012 and August 9th 2013, specifically for the purpose of this study. Participants were screened for physical conditions and psychiatric disorders. Participants reporting psychiatric disorders on the MINI plus interview were excluded from the study, as well as participants suffering from major physical illnesses. All controls completed the same questionnaires as the patients (ADHD-RS and PSQI). All controls signed an informed consent form before data collection.

### Neurofeedback treatment

Treatment of patients was identical to the methods published in Arns et al. (2012). In summary, all patients were assessed on a Quantitative EEG (QEEG) and an individualized neurofeedback treatment protocol was derived in line with the QEEG-informed decision rules reported in Arns et al. (2012). For this study only patients that were treated with an SMR or Theta/Beta protocol were included. In the SMR group all patients received a reward on 12–15 Hz at central locations (C3, Cz or C4); and the TBR group received mostly beta rewards outside the SMR frequency range (e.g., 20–25 Hz; 15–20 Hz) only at midline sites (Fz, FCz or Cz) in addition to theta inhibits. The locations for C3 and C4 for the SMR protocol were established using Transcranial Magnetic Stimulation (TMS) to individually localize the area where a visible response of the musculus abductor pollicis (thumb movement) was elicited (i.e., these were individualized “C3” and “C4” sites). In all protocols EMG inhibits were employed, meaning that the EMG (55–100 Hz) had to be kept below 5–10 μV.

Treatment was carried out by a masters level psychologist specialized in neurofeedback, supervised by the first author. Sessions took place 2–3 times a week, for 20–30 min provided in several 5-minute blocks, with 2 min pauses between successive blocks. The wireless Brainquiry PET 4.0 (Brainquiry B.V.) and BioExplorer software (CyberEvolution, Inc.) were used to provide visual feedback (bargraphs or neuropuzzles) and auditory feedback. Thresholds were set to achieve a 25–40% effective reinforcement. In addition for discrete SMR neurofeedback a time-above-threshold was set at 0.15–0.5 s.

## Analysis

Differences between groups were tested using One-Way ANOVA’s or non-parametric Mann-Whitney U test (gender). Furthermore, for quantifying the effects of neurofeedback on ADHD symptoms and sleep, a repeated measure ANOVA was used with within-subject factor Time (pre-treatment, mid-way treatment and post-treatment) and between subject factor Neurofeedback Protocol (SMR and TBR). In addition partial correlations covarying for age were used to further correlate changes in ADHD symptoms and sleep variables. Effect sizes (ES) reported are between-group or within-group pre-post-treatment Cohen’s D (*d*).

Mediator analysis will be performed in line with the MacArthur definitions and guidelines (Kraemer et al., 2002, 2008). The McArthur guidelines for mediator analyses require: (a) temporal precedence of the treatment; (b) an association between the mediator and treatment; and (c) a main effect of the mediator or an interaction between mediator and treatment (Kraemer et al., 2002, 2008). As mediator, the change in the significant sleep variables that change as a result of treatment will be correlated with improvement in inattention and hyperactivity/impulsivity.

Sleep Onset Latencies were log-transformed in order to meet a normal distribution, and for change across sessions a difference score (T_intake_–T_outttake_) was used rather than a percentage improvement score, since the latter resulted in non-normally distributed data.

## Results

Twenty-eight healthy controls (age: 21–64 yrs.; 13 male) and 52 patients with ADHD (age: 6–53 yrs; 37 male) were included in this study. For the comparison between controls and ADHD only adults will be included. For the within subject analysis of the effects of neurofeedback the whole ADHD group will be included.

### Healthy controls vs. adult ADHD

For the comparison between healthy adult controls (*n* = 28) and ADHD, only adults with ADHD (*N* = 19) were included and these groups did not differ in age (*p* = 0.990; *F* = 0.000; DF = 1, 46) and gender (*p* = 0.445; *Z* = −0.763). The adult ADHD group had significantly higher scores on the ADHD-RS inattention (*p* < 0.001; *F* = 345.246, DF = 1, 46), ADHD-RS hyperactivity/impulsivity (Hyp/Imp: *p* < 0.001; *F* = 36.108; DF = 1,46) and PSQI (*p* < 0.001; *F* = 47.090; DF = 1,46). Furthermore, on the PSQI, adults with ADHD reported a significantly longer SOL of 37 min compared to 14 min for controls (SOL: *p* = 0.011; *F* = 7.047; DF = 1, 46) and a significantly shorter sleep duration of 6.8 hrs. compared to 7.4 hrs. for controls (*p* = 0.014; *F* = 6.562; DF = 1,46), also see Table 1 for further details.

**Table 1:** **Differences between the control group and adult ADHD group on ADHD and sleep complaints**.

	Control group (N = 28)	Adult ADHD group (N = 19)	Cohen’s D
Age (yrs.)	34.1 (9.72)	34.1 (11.33)
ADHD-RS: Inattention	0.3 (0.67)	7.1 (1.76) ***	5.2
ADHD-RS: Hyp/Imp	0.9 (1.33)	4.5 (2.74) ***	1.7
PSQI	2.9 (1.18)	8.5 (4.05) ***	1.8
Sleep duration (hrs.)	7.4 (0.61)	6.8 (0.90) *	0.7
Sleep onset latency (min.)	13.8 (9.29)	37.2 (41.73) *	0.7

**Figure 2:**
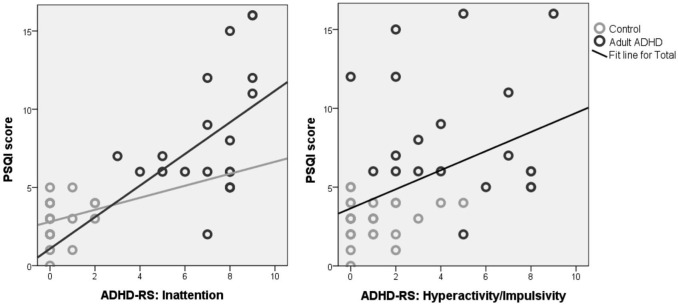
**Correlations between the PSQI score and inattention (left) and hyperactivity/impulsivity (right)**. Note the strong correlation for inattention, where the association between sleep problems (PSQI score) and ADHD symptoms was found for the whole group and also within the ADHD group, suggesting these almost seem to form a continuum from healthy controls (gray) to adults with ADHD (black), and for the whole group this association explained 59% of the variance for inattention. For Hyperactivity/Impulsivity the correlation was only significant for the whole group and not for the subgroups, suggesting this is only driven by group differences.

Correlations between ADHD complaints and sleep variables for the adult group yielded a significant correlation between age and sleep duration, hence partial correlations correcting for age were performed. Partial correlations with age as covariate yielded significant correlations between Inattention and PSQI score (*p* > 0.001; *r* = 0.789; DF = 44) for the whole group and performing this analysis separately for the ADHD group also resulted in a significant effect (*p* = 0.035; *r* = 0.499, DF = 16) but not for controls (*p* = 0.208; *r* = 0.250; DF = 25). Correlations between Inattention and Sleep Duration (*p* = 0.006; *r* = −0.401; DF = 44) and SOL (*p* = 0.004; *r* = 0.414; DF = 44) and between Impulsivity/Hyperactivity vs. PSQI score (*p* = 0.001; *r* = 0.464; DF = 44) and Sleep Duration (*p* = 0.027; *r* = −0.326; DF = 44) were only significant for the whole group, but not within the ADHD and control groups, suggesting these effects are driven only by the group differences. Figure 2 visualizes these correlations further.

Using the criterion from previous studies that a SOL latency of ≥30 min (both in children and adults) can be considered sleep onset insomnia (SOI), 29/51 (57%) of the whole sample of ADHD subjects vs. 5/28 (18%) of the controls met this definition, which was also significantly different between groups (*p* = 0.001; Chi-Square = 11.218). This analysis was conducted on the whole sample including the children, since the criterion used for SOI (>30 min) is the same for children and adults and thus independent of age (Van der Heijden et al., 2005; Van Veen et al., 2010).

### Neurofeedback treatment effects: SMR vs. TBR

Of the 51 ADHD patients included, 10 were treated with TBR Neurofeedback and 27 with SMR Neurofeedback (The remaining 14 patients were treated with combined SMR and TBR neurofeedback (*N* = 9) or only had intake data (*N* = 5)). There were no differences between these 2 groups on age, gender, ADHD-RS and PSQI measures (all *p* > 0.193), see Table 2. There were also no differences in the average number of sessions for the SMR (31 sessions) and TBR (29 sessions) groups (*p* = 0.656).

**Table 2:** **Baseline levels of ADHD and sleep complaints between the SMR neurofeedback treated group and TBR neurofeedback treated group and MSE (mean square error) and *p*-values**.

	SMR (N = 27)	TBR (N = 10)	MSE	*p*-value
Age (yrs.)	23.5 (14.5)	17.8 (12.8)	197.895	*p* = 0.280
ADHD-RS: Inattention	7.0 (1.8)	7.0 (1.6)	3.056	*p* = 0.955
ADHD-RS: Hyp/Imp	4.8 (2.6)	6.0 (2.1)	6.190	*p* = 0.193
PSQI	7.3 (4.1)	5.9 (2.5)	14.072	*p* = 0.322
Sleep duration (hrs.)	7.9 (1.8)	8.4 (1.3)	2.802	*p* = 0.517
Sleep onset latency (min.)	38.8 (35.7)	25.8 (13.8)	0.108^#^	*p* = 0.350^#^
Number of sessions	31.5 (13.0)	29.5 (7.8)	138.871	*p* = 0.656

*Note that ^#^ means statistics based on log-transformed data*.

A repeated measures ANOVA with within-subject factor Time (pre-treatment, mid-way treatment and post-treatment) and between-subject factor Protocol (SMR vs. TBR) yielded significant Time effects (improvement) for Inattention (*p* < 0.001; *F* = 82.631; DF = 2,34; *d* = 2.6), Hyp/Imp (*p* < 0.001; *F* = 51.529; DF = 2,34; *d* = 1.8), PSQI score (*p* > 0.001; *F* = 11.417; DF = 2, 34; *d* = 0.9) and no significant Time X Protocol nor a main effect of Protocol, suggesting that both protocols had similar effects on main ADHD symptoms and PSQI score. For sleep duration no main effect of Time or Protocol, or Time X Protocol interaction were found.

**Figure 3:**
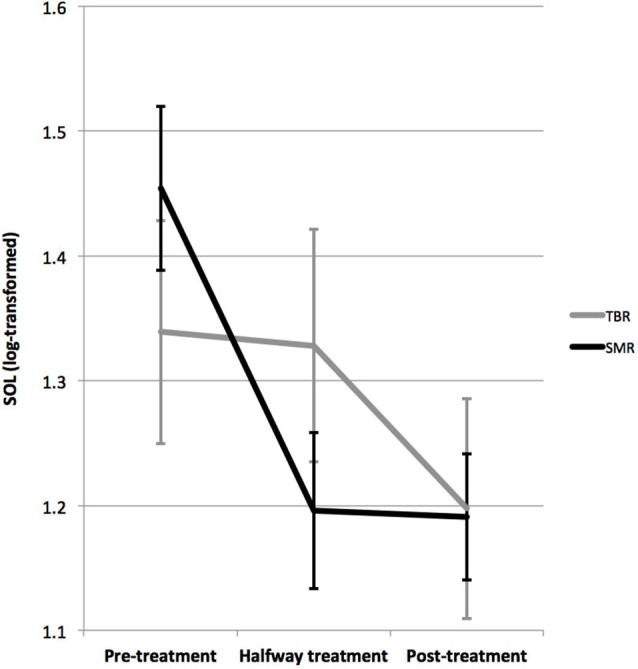
**This figure demonstrates the interaction between the SMR and TBR treated groups on SOL.** The SMR treated group demonstrated a decrease in SOL from pre-treatment to post-treatment from 40 to 19 min, where this effect was most pronounced within the first half of treatment (*p* < 0.001), whereas for TBR this effect was not significant (*p* = 0.921) and only a significant pre-treatment to post-treatment (26 to 19 min) effect was found (*p* = 0.036). The time effects were significant for both groups.

For SOL a near significant Time X Protocol interaction (*p* = 0.076; *F* = 2.795; DF = 2, 32) and a Time effect (*p* = 0.002; *F* = 7.365; DF = 2, 32) were found, but not of Protocol (note that for 2 patients in the SMR group there were missing data explaining the lower DF values). Figure 3 visualizes this interaction further. As can be seen, the time effect (post-minus pre-treatment) is substantially larger for the SMR group than it is for the TBR group. This was further confirmed by paired sample t-test that found a significant decrease in SOL from pre-treatment to post treatment for TBR (*p* = 0.036) and SMR (*p* < 0.001), but only a significant decrease from pre-treatment to halfway treatment for SMR (*p* < 0.001) and not for TBR (*p* = 0.921).

Repeating the analyses separately for SMR and TBR, yielded a significant time effect for each: For SMR (*p* < 0.001; *F* = 12.337; DF = 2, 23; *d* = 0.9), where SOL decreased from 40.1 min pre-treatment to 19.1 min post-treatment; for TBR (*p* = 0.036: *F* = 5.153; DF = 2, 8; *d* = 0.5) where SOL decreased from 25.8 min to 18.8 min post-treatment. Repeating this analysis in children only or adults only resulted in similar effects and a similar trend for interaction.

#### Mediator analysis

Age did not correlate with change in inattention (*p* = 0.980, *r* = 0.004) and hyperactivity/impulsivity (*p* = 0.879, *r* = −0.026), and there was no difference between males and females in change in inattention (*p* = 0.636) and hyperactivity/impulsivity (*p* = 0.885) suggesting these variables do not moderate treatment outcome to neurofeedback treatment.

Given the above interaction between SOL and treatment protocol, mediator analyses were conducted for TBR and SMR separately.

The change in SOL from pre- to post-treatment was larger for the SMR group (21 min) as compared to the TBR group (7 min), however this difference was not significant (*p* = 0.132; *F* = 2.378; DF = 1, 36), and this change in SOL occurred earlier for the SMR group as compared to the TBR group (see Figure 3), thus the criterion of temporal precedence is fulfilled.

**Figure 4:**
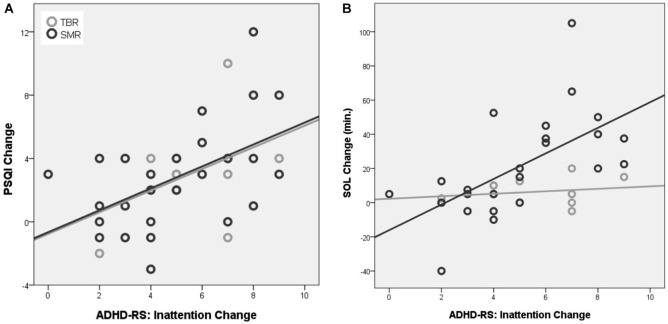
**Correlation between the change in ADHD-RS inattention and change in PSQI (A) and change in SOL (B)**. Note that only for the SMR treated group a significant association between improvement on PSQI change and inattention and SOL and inattention was observed that explained 34% and 39% of the variance respectively.

A significant correlation between the change in inattention and change in PSQI score was found for the SMR group (*p* = 0.006; *r* = 0.518; DF = 27) and not for the TBR group (*p* = 0.206; *r* = 0.437; DF = 10), also see Figure 4A. No correlation was found for change in hyperactivity/impulsivity. A significant correlation between the change in inattention and change in SOL was found for the SMR group (*p* = 0.001; *r* = 0.625; DF = 26) and not for the TBR group (*p* = 0.653; *r* = 0.163; DF = 10), also see Figure 4B. No correlation was found for change in hyperactivity/impulsivity (*p* > 0.358). Therefore, the criterion of association is also met.

When repeating the repeated measures ANOVA for inattention, including SOL change as a between subject factor, did not result in a main effect of SOL change (*p* = 0.880; *F* = 0.541; DF = 19,10), a Time X Protocol X SOL change (*p* = 0.649; *F* = 0.778; DF = 10,20) or Protocol X SOL Change interaction (*p* = 0.874; *F* = 0.345; DF = 5,10), whereby the third criterion for mediation is officially not met.

### Learning

**Figure 5:**
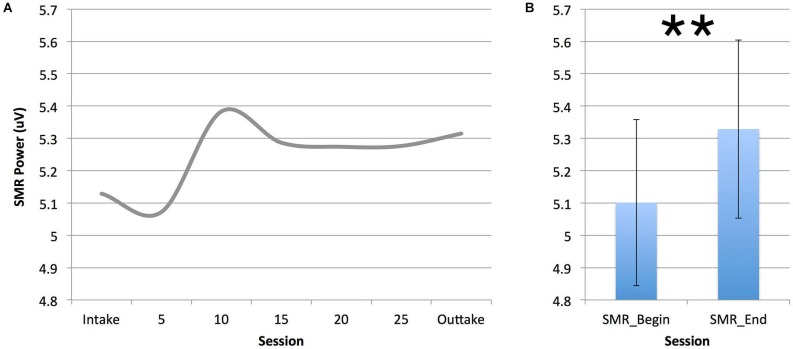
**This figure demonstrates the increase in SMR power across training sessions (A)**. Note that the SMR neurofeedback group was able to increase SMR power after 10 sessions, confirmed by comparing SMR power between the beginning and end of treatment (**p ≤ 0.01).

Figure 5 below depicts the SMR power during the first 10 min of neurofeedback intake, outtake and sessions 5, 10, 20 and 25 for the group treated with SMR neurofeedback. As can be seen SMR power during sessions starts to increase at session 10. A repeated measures ANOVA with average SMR at the beginning (intake and session 5) and average SMR at the end (session 15 to outtake) yielded a significant effect of time (*p* = 0.010; *F* = 7.663; DF = 1, 26; *d* = 0.2), also see Figure 5B. Of the 27 people that underwent SMR neurofeedback, 20 (74%) were able to increase their SMR from begin to end. Learners had a smaller decrease in PSQI score (*p* = 0.024, *F* = 5.801; DF = 1,26) as compared to non-learners. No differences were found for inattention, hyperactivity/impulsivity, SOL and sleep duration.

The TBR group was too small to conduct proper statistics. Visually, for beta a U-shaped distribution over sessions was found, where the decrease in beta from intake to session 15 paralleled the decrease in EMG, and when EMG remained flat beta increased from session 15 to outtake.

### *Post-hoc* tests

In the SMR neurofeedback group, for 8 of the 27 patients a theta inhibit was used, whereas for the other 19 patients only SMR was trained. Repeating the above repeated measure ANOVA’s did not yield any interactions between these 2 groups. Of the 27 patients treated with SMR neurofeedback, 12 were treated at C4 and 13 were treated at C3 (the remaining 2 were trained at Cz), also when repeating the above analysis with left vs. right SMR neurofeedback yielded no interactions with laterality.

## Discussion

In this study we found that adults with ADHD, reported more sleep problems (PSQI score), a shorter sleep duration (36 min less sleep on average) and a longer sleep onset latency (SOL: 23 min more to fall asleep) than adults without ADHD. When using a cut-off of 30 min for SOL (Smits et al., 2001; Rybak et al., 2007) we found that 57% of the ADHD adults and children had sleep-onset insomnia (SOI) as compared to 18% of the control group, which is in line with previous studies that reported 72–78% of SOI in ADHD adults and children (Van der Heijden et al., 2005; Van Veen et al., 2010). Furthermore, for the adult group of ADHD patients and controls, strong correlations were found between reported sleep problems and inattention, explaining 59% of the variance. This correlation was also significant in the ADHD group, and had the same direction (albeit non-significant) in the control group, suggesting this relationship is not simply driven by group differences. Figure 2 visualizes this association further, and it looks like the relation between reported sleep problems and inattention constitutes a continuum, where problems of inattention are strongly related to reported sleep problems. This overall PSQI score likely reflects a multitude of possible sleep problems that are likely to affect both Process S directly (e.g., RLS, Sleep apnea) as well as via Process C (delayed circadian phase), therefore the strength of this effect mainly suggests sleep disruptive processes explaining impaired attention, albeit this does not implicate *specific* effects.

Both SMR and TBR neurofeedback had similar clinical effects on inattention, impulsivity/hyperactivity and reported sleep problems in this study. On the other hand, SMR neurofeedback had its most specific effect on decreasing SOL (specifically in the first half of treatment, see Figure 3), further demonstrated by the strong correlations between inattention improvement and SOL improvement (39% explained variance; see Figure 4B) suggesting the change in SOL could be considered a mediator of treatment response for SMR neurofeedback. These data are in agreement with the proposed working mechanism as presented in the introduction, and suggest that the effects of SMR neurofeedback could results in increased sleep spindle density, which would explain the decreased SOL. The association between SOL improvement and behavioral improvement were most specifically found for inattention but not for hyperactivity/impulsivity, which is in line with our earlier proposal where inattention is a direct result of sleep problems (vigilance dysregulation), whereas the hyperactivity and impulsivity are considered to be vigilance autostabilization behavior, or an indirect compensatory mechanism (Arns and Kenemans, 2014).

The McArthur guidelines for mediator analyses require: (a) temporal precedence of the treatment; (b) an association between the mediator (SOL change) and treatment (inattention change); and (c) a main effect of the mediator or an interaction between mediator and treatment (Kraemer et al., 2002, 2008). The mediator analysis fulfilled criteria (a) and (b); but not criterion (c) (a main effect for SOL change or a Protocol X SOL Change interaction), thereby formally not meeting the definition of the McArthur guidelines. However, since this study was not a randomized controlled trial, the TBR group also demonstrated improvements in SOL (albeit not correlated to improvement on inattention) and the limited sample size of the TBR group might explain this lack of a main effect or interaction with SOL change. Therefore, future randomized controlled trials, such as for example the trial by the Collaborative Neurofeedback Group (The Collaborative Neurofeedback Group, 2013), should conduct such mediator analysis to further demonstrate that clinical effects of SMR neurofeedback are mediated by SOL.

For TBR neurofeedback no association between clinical improvement and change in SOL or PSQI were found. Given that patients treated with TBR neurofeedback were mainly trained at midline sites (Fz, FCz or Cz) and at frequencies above the SMR frequency band and the clinical effects were the same, suggests at least a differential effect of these two neurofeedback protocols. Furthermore, this suggests that the proposed working mechanism of SMR neurofeedback as discussed in the introduction and in Arns and Kenemans (2014) might not generalize to TBR neurofeedback. Along these lines, maybe the effects of TBR neurofeedback can be better explained by the model put forward by Gevensleben and colleagues (Gevensleben et al., 2012), where the effects of TBR and SCP neurofeedback are mainly explained by learned self-regulation over brain activity associated with attention (decreased theta and increased beta as an indication of a desynchronized brain state).

For the SMR group a significant increase in SMR power was observed across training sessions, demonstrating that indeed learning took place on SMR power and 74% of patients were able to increase their SMR across sessions. However, there were no differences in clinical outcome between learners and non-learners, only an effect on PSQI score, where learners had a smaller decrease in PSQI score. Therefore, the question also arises how SMR neurofeedback really exerts its clinical effect. In most studies the assumption is that uni-directional training, in this case SMR uptraining, is required for clinical effects. However, in Slow Cortical Potential (SCP) neurofeedback bidirectional training is employed in order to learn patients to self-regulate the SCP. In a previous study we investigated bidirectional SMR neurofeedback, and found that some people learn to control their SMR mostly by upregulating SMR, whereas another group learned to control SMR mostly by downregulating SMR (Kleinnijenhuis et al., 2008). In Arns and Kenemans (2014) it was also stated that *… SMR neurofeedback is not about increasing the EEG power in a specific frequency range, but rather about regulating activity within a functional network (reticulo- thalamocortical network, also see Section 2.6), thereby increasing the synaptic strength within this network, resulting in long-term potentiation (LTP) which increases synaptic sensitivity and the probability of future activation in this network…*” Arns and Kenemans (2014). In this view it could thus be that some patients are more successful in up- and others in down-regulating SMR, and either approach resulting in increased sleep spindle density. Future studies should investigate this in more detail by employing bi-directional SMR training in patient populations.

Limitations of the study include: (1) In this study we did not assess Dim Light Melatonin Onset (DLMO) and our results on SOI are based on self-report using the PSQI whereby we could not formally define SOI in line with (Smits et al., 2001; Van Veen et al., 2010). However, interestingly our percentage of 56% SOI in adults with ADHD seems in line with previous studies. (2) In this study only self-report of sleep parameters was used. Future studies should further investigate these effects with more objective measures such as actigraphy, polysomnography or DLMO. (3) The mediator analyses did not yield a main effect nor an interaction with treatment, whereby formally based on the MacArthur guidelines, baseline SOL cannot be regarded as a mediator (Kraemer et al., 2002, 2008). The lack of this significant interaction is possibly explained by the comparison of two active conditions (SMR and TBR) and not an active vs. placebo condition, hence future randomized controlled trials should more specifically investigate this.

Concluding, in this study we found a clear continuous relationship between self-reported sleep problems (PSQI) and inattention in adults with- and without-ADHD, that explained 59% of the variance, prompting researchers and clinicians to pay more attention to identify sleep problems in patients suspected of ADHD to—in line with DSM 5—rule out other causes of inattention. If confirmed, such sleep problems might require treatment first, before treatment is focused on ADHD treatments in line with other studies (Cortese et al., 2013; Miano et al., 2012). TBR neurofeedback resulted in a small reduction of SOL, this change in SOL did not correlate with the change in ADHD symptoms and the reduction in SOL only happened in the last half of treatment, suggesting this is an effect of symptom improvement not specifically related to TBR neurofeedback. SMR neurofeedback specifically reduced the SOL and PSQI score, and the change in SOL and change in PSQI correlated strongly with the change in inattention, and the reduction in SOL was achieved in the first half of treatment, suggesting the reduction in SOL mediated treatment response to SMR neurofeedback. Clinically, TBR and SMR neurofeedback had similar effects on symptom reduction in ADHD (inattention and hyperactivity/impulsivity), therefore these results suggest differential effects and different working mechanisms for TBR and SMR neurofeedback in the treatment of ADHD. Future studies should investigate and replicate these findings in more controlled studies using more objective measures of SOL and sleep duration.

## Author contributions

MA initiated this study, conducted the statistical analyses and initiated a first version of the manuscript. Ilse Feddema was responsible for the data collection and management, and assisted in writing the manuscript. J. Leon Kenemans assisted in writing the manuscript.

## Disclosures

Martijn Arns reports research grants and options from Brain Resource Ltd. (Sydney, Australia), equipment support from Deymed Diagnostic and is a paid consultant for United BioSource Corporation (UBC), Bracket and Vivatech and is a co-inventor on 3 patent applications (A61B5/0402; US2007/0299323, A1; WO2010/139361 A1) related to EEG, neuromodulation and psychophysiology, but does not own these nor receives any proceeds related to these patents.

## Conflict of interest statement

Martijn Arns reports research grants and options from Brain Resource Ltd. (Sydney, Australia), equipment support from Deymed Diagnostic and is a paid consultant for United BioSource Corporation (UBC), Bracket and Vivatech and is a co-inventor on 3 patent applications (A61B5/0402; US2007/0299323, A1; WO2010/139361 A1) related to EEG, neuromodulation and psychophysiology, but does not own these nor receives any proceeds related to these patents.
